# Reemerging Murine Typhus, Japan

**DOI:** 10.3201/eid1005.030697

**Published:** 2004-05

**Authors:** Satoshi Sakaguchi, Ichiki Sato, Hiroaki Muguruma, Hiroaki Kawano, Yoshito Kusuhara, Seiji Yano, Saburo Sone, Tsuneo Uchiyama

**Affiliations:** * The University of Tokushima Graduate School of Medicine, Tokushima, Japan; † Sato Clinic, Tokushima, Japan

**Keywords:** Murine typhus, serological diagnosis, reemergence, *Rickettsia typhi*, Japan

**To the Editor:** Murine typhus is an arthropod-borne infectious disease caused by *Rickettsia typhi*, which is distributed widely around the world (*1*–*4*). In Japan, tsutsugamushi disease occurs most frequently in persons infected with rickettsioses (*5*). Spotted fever caused by *R. japonica* also occurs in the southwestern part of Japan (*6*,*7*). In the 1940s and 1950s, many murine typhus cases were reported in Japan. These diagnoses were made according to the clinical features of the illness and the reactivity of the serum samples to OX19 in Weil-Felix tests. A few cases were diagnosed on the basis of symptoms exhibited by animals infected with isolated rickettsiae and complement fixation tests, in addition to results of the Weil-Felix tests. The Weil-Felix test is useful for preliminary screening of rickettsiosis; however, the reaction could indicate epidemic typhus or spotted fever in some cases. Since 1958, only three murine typhus cases have been reported in Japan (*8*). In these cases, no serologic tests for epidemic typhus were conducted. Serum sample from patients with epidemic typhus and murine typhus frequently possess serologic cross-reactivity to *R. typhi* and *R. prowazekii*, respectively (*9*). Thus, the possibility of epidemic typhus could not be excluded definitively in these cases.

On May 4, 2003, a 56-year-old man living in Tokushima, Japan, sought medical care; he had a temperature of 39.1°C and exanthema on the trunk and the upper limbs. No surface lymph nodes were palpable. He was treated with lincomycin and cefditoren pivoxil with no improvement. On day 3, the patient informed caregivers that he had been in a bamboo grove on days 1 and 11 before the onset of symptoms. C-reactive protein of the serum sample collected on day 3 was positive (= 7.6 mg/dL). From this finding, spotted fever was suspected; the disease is endemic in Tokushima. On day 4, the exanthema had spread systemically, and treatment with minocycline was started, which led to a gradual decrease in fever and rashes. The patient was admitted to the Tokushima University Hospital on day 6 of the illness for diagnosis and further treatment.

Serum samples were collected from the patient on days 5, 6, 9, 20, and 34. Indirect immunoperoxidase tests on the serum samples for tsutsugamushi disease, spotted fever, murine typhus, and Q fever on day 5 of the illness were negative for immunoglobulin (Ig) G and IgM antibodies (<1:40). Weil-Felix tests on the serum samples on days 5 and 9 of the illness were negative for OX2, OX19, and OXK. Indirect immunofluorescence of the serum samples on days 6, 9, 20, and 34 of the illness was conducted by using strains 18 and Wilmington of *R. typhi,* and the strain Breinl of *R. prowazekii* as typhus group rickettsiae; and the strain YH of *R. japonica*, the strain Malish 7 of *R. conorii*, and the strain Tick of *R. montanensis* as the spotted fever group rickettsiae. All serum samples tested for the rickettsiae showed an IgM titer of 1:200. On the other hand, the IgM titers of these serum samples, to the *Orientia tsutsugamushi* were <1:20. For the IgG antibodies of these serum, spotted fever group rickettsiae were negative (<1:20). However, the typhus group rickettsiae were positive for IgG antibodies. Among the typhus group rickettsiae, strain 18 of *R. typhi* had the highest elevated titers. The titers to the sera on days 6, 9, 20, and 34 of illness were 1:80, 1:160, 1:160, and 1:80, respectively. Another strain of *R. typhi*, the strain Wilmington, had lower titers of 1:40, 1:80, 1:80, and 1:40, on days 6, 9, 20, and 34 of the illness, respectively. This could have occurred because strain 18 may be a closer antigenic relation of the causative agent than is the strain Wilmington. *R. prowazekii* demonstrated the lowest IgG titers among typhus group rickettsiae for these serum samples, <1:20, 1:20, 1:40, and 1:20, on days 6, 9, 20, and 34 of the illness, respectively. These results suggested that the disease was murine typhus.

To demonstrate more detailed antigenic reactivity, Western immunoblotting of rickettsiae was conducted by using a serum specimen from day 20. All of the rickettsiae were reactive to the serum to various extents. The serum reacted to the ladderlike lipopolysaccharide of *R. typhi* and *R. prowazekii*; the antigenicity of rickettsial lipopolysaccharide is group-specific. As expected from the immunofluorescence data, no reaction was demonstrated to the lipopolysaccharide of spotted fever group rickettsiae, *R. japonica* and *R. montanensis*, although trace cross-reactivity, mainly to *rOmpB*, was shown. Thus, typhus group rickettsiosis was suspected for this case on the basis of these data. Compared to the trace reaction of spotted fever group rickettsiae to *rOmpB*, a stronger, but still weak, reaction was detected to the heat-labile state of rOmpB of *R. prowazekii,* and an extremely strong reaction was demonstrated to the heat-labile and heat-stable states of *rOmpB* of *R. typhi*. These results strongly suggested that the disease was murine typhus.

To confirm this diagnosis, we conducted absorption tests as described previously (*10*). The patient serum collected on day 20 showed complete absorption by the homologous antigen, the purified *R. typhi* strain 18, demonstrating no reaction to *R. typhi* or to *R. prowazekii* by immunofluorescence. However, the serum showed incomplete absorption by the heterologous antigen, the purified *R. prowazekii*, demonstrating no reactivity to *R. prowazekii* but some reactivity to *R. typhi*. These tests confirmed the diagnosis of murine typhus.

Murine typhus has never been reported in Japan after the 1950s, except for the three suspected cases and this case. Although other undiagnosed cases may have occurred, they appear to be few; many febrile cases of exanthema have been examined for various rickettsioses, especially after spotted fever was diagnosed in Japan in 1984. Murine typhus may have reemerged because of the recent increase of black rats, *Rattus rattus,* in Japan. This patient mentioned that he had captured a rat and disposed of the carcass about a week before the onset of symptoms. Infection could have resulted at that time from contamination with feces of infected fleas such as the oriental rat flea, *Xenopsylla cheopis.* Historical review indicates that this is the first complete serologic diagnosis of a murine typhus case in Japan.

## References

[1] Parola P, Vogelaers D, Roure C. Janbon, Raoult D. Murine typhus in travelers returning from Indonesia. Emerg Infect Dis. 1998;4:677–80. 10.3201/eid0404.980423 9866749 PMC2640266

[2] Azad AF, Beard CB. Rickettsial pathogens and their arthropod vectors. Emerg Infect Dis. 1998;4:179–86. 10.3201/eid0402.980205 9621188 PMC2640117

[3] Walker DH. Advances in understanding of typhus group rickettsial infections. In: Kazar J, Toman R, editors. Rickettsiae and rickettsial diseases. Bratislava, Slovak Republic: VEDA Press; 1996. p.16–25.

[4] Azad AF, Radulovic S, Higgins JA, Noden BH, Troyer MJ. Flea-borne rickettsioses: some ecological considerations. Emerg Infect Dis. 1997;3:319–28. 10.3201/eid0303.970308 9284376 PMC2627639

[5] Kawamura A Jr, Nogami S. Seasonal prevalence of the disease in Japan. In: Kawamura A Jr, Tanaka H, Tamura A, editors. Tsutsugamushi disease. Tokyo, Japan: University of Tokyo Press; 1995. p.10–20.

[6] Mahara F, Koga K, Sawada T, Taniguchi F, Shigemi T, Suto Y, The first report of rickettsial infections of spotted fever group in Japan; three clinical cases. [in Japanese]. Kansenshogaku Zasshi. 1985;59:1165–72. 3938467 10.11150/kansenshogakuzasshi1970.59.1165

[7] Uchida T, Uchiyama T, Kumano K, Walker DH. *Rickettsia japonica* sp. nov., the etiological agent of spotted fever group rickettsiosis in Japan. Int J Syst Bacteriol. 1992;42:303–5. 10.1099/00207713-42-2-303 1581190

[8] Takagi K, Iwasaki H, Kishi S, Nakamura T, Takada N, Ueda T. Murine typhus infected in Oku-etsu area, Fukui Prefecture. [in Japanese]. Kansenshogaku Zasshi. 2001;75:341–4. 11357325 10.11150/kansenshogakuzasshi1970.75.341

[9] La Scola B, Raoult D. Laboratory diagnosis of rickettsioses: current approaches to diagnosis of old and new rickettsial diseases. J Clin Microbiol. 1997;35:2715–27. 9350721 10.1128/jcm.35.11.2715-2727.1997 PMC230049

[10] La Scola B, Rydkina L, Ndihokubwayo JB, Vene S, Raoult D. Serological differentiation of murine typhus and epidemic typhus using cross-adsorption and Western blotting. Clin Diagn Lab Immunol. 2000;7:612–6. 10882661 10.1128/cdli.7.4.612-616.2000 PMC95923

